# E-cadherin Single Nucleotide Variants Are Associated with Increasing Susceptibility to Periodontitis

**DOI:** 10.1055/s-0044-1791683

**Published:** 2024-11-07

**Authors:** Nadia M. Kazem, Ali A. Abdulkareem, Paul R. Cooper, Michael R. Milward

**Affiliations:** 1 Department of Periodontics, College of Dentistry, University of Baghdad, Bab Al Mudam, Baghdad, Iraq; 2 Department of Oral Sciences, Faculty of Dentistry, Sir John Walsh Research Institute, University of Otago, Dunedin, New Zealand; 3 School of Dentistry, University of Birmingham, Birmingham, United Kingdom

**Keywords:** periodontitis, E-cadherin, biomarkers, gene polymorphism

## Abstract

**Objectives** To investigate the association of *E-cadherin* single nucleotide polymorphisms (SNPs) with periodontitis and the potential of these SNPs for identifying susceptibility to periodontitis.

**Materials and Methods** Periodontal clinical parameters were recorded followed by collecting venous blood for DNA extraction. Polymerase chain reaction was used to amplify target segments of the *E-cadherin* gene. Determination of the genotype and allele frequencies was performed using Sanger sequencing. All statistical analyses were performed using GraphPad Prism (version 9) using a statistically significant difference of *p* < 0.05.

**Results** A total of 207 participants were recruited into two groups of healthy controls ( *n* = 105) and cases diagnosed with periodontitis stage 2 or 3, grade B or C ( *n* = 102). Analyses indicated that the genotypes and alleles of rs3743674 and rs5030625 *E-cadherin* SNPs were significantly associated with periodontitis. Results from a binary regression model suggested that the presence of these SNPs may indicate susceptibility to periodontitis and increase the rate of progression. Linkage disequilibrium analysis indicated that *E-cadherin* variants rs3743674 and rs5030625, and rs10272115 and rs16260 were correlated in a nonrandom manner ( *r* ^2^ = 0.638 and 0.495, respectively).

**Conclusion** E-cadherin gene variants, rs3743674 and rs5030625, were associated with the periodontitis phenotype. These biomarkers may identify individuals susceptible to periodontitis and the rate of disease progression.

## Introduction

Periodontitis is a multifactorial inflammatory disease with alternating episodes of remission and exacerbation leading to progressive destruction of periodontal tissues, resorption of alveolar bone, and potential for tooth loss in the later stages. 1 Despite efforts of health care institutes worldwide, periodontitis is still ranked 11th among chronic diseases affecting adults with a prevalence between 20 and 50%. 2 3 This is partly attributed to the global lack of knowledge about the nature and consequences of this disease. 4 In addition, the involvement of multiple factors such as genetic, environmental, and lifestyle risk factors represents obstacles to prevention, diagnosis, and management strategies. 5

Undoubtedly, an increasing bacterial load of dental plaque biofilm around the gingival margin is the main driver for initiation of gingivitis which progresses to periodontitis in susceptible individuals characterized by an exaggerated immune response on interaction with a dysbiotic subgingival microbiota. 6 7 In addition, the degree of severity and rate of progression of periodontitis are also variable among patients and this is modulated by risk factors. 8 A genetic component in the pathogenesis of periodontitis has long been highlighted, and this indicates an individual predisposition to the disease. Single nucleotide polymorphisms (SNPs) are differences in single nucleotides within DNA and are the most frequent genetic variation affecting humans. 9 On average, each individual's genome exhibits 4 to 5 million SNPs with more than 600 million SNPs currently being identified. Many studies have been conducted and systematically reviewed to determine associations between periodontitis and a range of SNPs that affect immune system components, and gene variants of interleukins (ILs) have been extensively investigated. 10 11

Integrity of the sulcular/pocket epithelial lining is maintained by the coherence of epithelial cells primarily provided by adherens junctions–mediated intercellular junctions. The basic structural unit of adherens junctions is a glycoprotein known as E-cadherin which is a calcium-dependent molecule. 12 The cytoplasmic domain of E-cadherin integrates with the cytoskeleton via anchoring proteins, including p120 catenin, β-catenin, and α-catenin, forming a belt-like intercellular structure which provides mechanical strength to the cellular attachment. 13 Evidence from *in vitro* 14 and *in vivo* 15 studies have now indicated that the expression and level of E-cadherin are decreased in epithelial cells exposed to periodontal pathogens. Subsequently, this results in loss of epithelial phenotype and disintegration of tissue integrity, and this ulceration is a whole mark of periodontitis pathogenesis and allows the spread of potent periodontal pathogens into the underlying tissues and systemic circulation. 16 A recent study has demonstrated a significant increase of salivary E-cadherin level in periodontitis cases and this points to disintegration of the cellular attachment apparatus due to the release of the E-cadherin molecule. 17 The role of *E-cadherin* SNPs has previously been demonstrated in many diseases, and this mechanism is highlighted as being responsible for tissue instability and vulnerability of E-cadherin to pathologic events. 18

Thus far, the pathogenic mechanism responsible for periodontitis is not yet fully defined; however, recent literature suggests common features between periodontitis and malignancy include activation of the Wnt/β-catenin pathway responsible for loss of epithelial phenotype associated with E-cadherin downregulation. 19 Genetic variants of *E-cadherin* reportedly play a role in cancer predisposition 18 and can predict the survival outcome of patients with breast cancer. 20 Consequently, we hypothesized that E-cadherin SNPs could be associated with increasing susceptibility to periodontitis. Therefore, this study was conducted to investigate this possible association and the potential of these genetic variants to predict periodontitis in an Arab Iraqi population.

## Materials and Methods

### Study Design and Participants

Participants in this case–control study (1:1 allocation) were recruited on a consecutive basis from the patients attending the teaching clinics at the Department of Periodontics, College of Dentistry, University of Baghdad, and the Iraqi National Blood Bank, Baghdad, Iraq, from September to December 2022. Ethical principles stated by the latest version of Declaration of Helsinki for human research in 2013 were followed for all procedures. Prior to conducting the study, ethical approval was obtained from the local Ethics Committee, College of Dentistry, University of Baghdad (ref. no. 647, project no. 647622, date: September 13, 2022). The aim, nature, and methods of the study were clearly described to each participant, a chance for any questions to be answered, before signing the consent form. All subjects were primarily assessed for their eligibility to join the study. The recruited participants were divided into periodontally healthy subjects (controls) and patients with periodontitis (cases) as determined by the assessment described later.

### Inclusion and Exclusion Criteria

Subjects recruited to this study were systemically healthy with no history of any disease that was considered as a risk factor for periodontitis, such as diabetes mellitus and cardiovascular diseases, or any disease related to *E-cadherin* SNPs, including cancers, ulcerative colitis, Crohn's disease, and asthma. All subjects were older than 18 years, had a minimum of 20 teeth, not taking any medication within the last 3 months, and exhibited a normal body mass index (18.5–24.9). The ethnic group included in this study was of Arabic Iraqi individuals of both sexes. Healthy periodontium was defined by the presence of bleeding on probing (BOP) in <10% of total sites with probing pocket depth (PPD) not exceeding 3 mm and no evidence of clinical attachment loss (CAL), that is, intact periodontium. 21 The diagnosis of periodontitis was confirmed when two or more teeth exhibited interdental CAL or when CAL ≥3 mm was confined to the buccal or lingual sites associated with PPD >3 mm. 1 Confirmed periodontitis cases were further defined according to stage and grade. 1 Periodontitis cases of any stage and grade were included in this work. The stage was defined by the severity of bone loss at the worst site relative to the root according to the criteria set by the American Academy of Periodontology and European Federation of Periodontology. 1 The grade was estimated by dividing percentage of bone loss at the worst site by the age, if the result was 0.25 to 1.0, then the grade was B and grade C was given when the result was >1.0. Reasons for exclusion were the risk factors including diabetes mellitus, smoking, pregnancy, consumption of antibiotics, and/or anti-inflammatory medication in the last 3 months and subjects not willing to consent.

### Periodontal Parameters

First, the patients were initially screened depending on the criteria for periodontal health and periodontitis. Then, each patient received full-periodontal charting including BOP, PPD, and CAL which were recorded at six sites, distal, middle, and mesial aspects of the labial/buccal and palatal/lingual surfaces, per tooth. All periodontal parameters were recorded by the same calibrated examiner by using a UNC-15 periodontal probe. After gently inserting the probe to the depth of the sulcus/pocket, BOP was scored as 0 or 1 depending on the absence or presence of bleeding, respectively. Simultaneously, the distances from the gingival margin and cementoenamel junction to the base of the sulcus/pocket were measured and recorded as PPD and CAL, respectively. For examiner calibration, the criteria of all clinical periodontal parameters were discussed first between the main investigator (N.M.K.) and expert periodontist (A.A.A.). Then, calibration sessions were conducted as previously described on five patients with a minimum of 2 hours between the measurements of the examiners. 22 These sessions were repeated until the intraclass correlation coefficient was ≥ 90% for CAL and PPD, while the accepted level of consistency for BOP was ≥ 70% which was determined by using the kappa coefficient test.

### Blood Sampling and Preparation of DNA

A 2 mL of venous blood was withdrawn from each participant using a 5-mL disposable syringe with a 20-gauge needle, from the antecubital fossa by venipuncture under aseptic conditions. Samples were transferred into EDTA tubes (Medicalet, Shenzhen, China) and were stored at −4°C until DNA was extracted. The DNA was isolated from blood samples using the total DNA extraction kit (ABIOpure, United States) following the manufacturer's instructions. Blood samples were thoroughly mixed for 15 minutes then from each 1.5 mL microcentrifuge tube, 20 µL of proteinase K solution was dispensed, and 200 µL of blood was added and mixed. Lysate buffer was added to the tube, mixed, and incubated in a water bath at 56°C for 30 minutes. This was followed by adding 200 μL of absolute ethanol to the thoroughly mixed samples. The mixture was transferred to the minicolumn assembly and sequentially centrifuged (Fisher Scientific, United States) for 1 minute at 8,000 rpm, adding wash buffers as instructed by the manufacturer. After removing residual wash buffer by centrifugation for 1 minute at 10,000 rpm, buffer AE (100 μL) was added, incubated for 1 minute, and finally centrifuged at 5,000 rpm for 5 minutes. The concentration of the extracted DNA was determined by mixing 1 µL of the DNA with diluted Quantifluor Dye and analysis in a Quantus Fluorometer (Promega, United States).

### PCR Amplification

E-cadherin (CDH1) primers, forward 3′-5′ > TGTAAAACGACGGCCAGTCCCGACTTGTCTCTCTACA and reverse 3′-5′ > CAGGAAACAGCTATGACTCAGGACCCGAACTTTCT, were obtained from Macrogen (Seoul, Republic of Korea) in a lyophilized form. These primers were prepared to obtain a final working solution of 10 pmol/μL. After confirming DNA quality, polymerase chain reaction (PCR) amplifications were performed with 25 μL volumes containing 12.5 µL GoTaq Green Master Mix, 1 µL of each primer, 8.5 µL nuclease-free water, and 2 µL of template DNA. PCR cycling was performed with the PCR express (Thermal Cycler, BioRad, United States) using the following thermal cycling conditions: initial denaturation at 94°C for 4 minutes followed by 30 cycles of denaturation at 94°C for 30 seconds; annealing at 55, 58, 60, 65°C for 30 seconds; and extension at 72°C for 30 seconds. A final extension incubation of 7 minutes at 72°C was performed and the reaction was stopped by incubation at 4°C for 10 minutes.

### Agarose Gel Electrophoresis

This step was performed to confirm PCR product amplification. The gel was prepared by combining 1.5 g (1.5%) agarose (Promega) with 100 mL of 1X Tris-acetate-EDTA (TAE) buffer and heating until all particles were dissolved. Then, 1 μL of ethidium bromide (10 mg/mL) was added to the agarose solution which was allowed to cool down to 50 to 60°C. At this stage, the solution was poured into a predammed gel tray with a well-forming comb and allowed to solidify at room temperature for 30 minutes. The gel was fully immersed in the gel electrophoresis tank containing 1X TAE-electrophoresis buffer. A 5 μL of PCR product and ladder were carefully loaded into the wells, and an electric current at 100 V/50 mAmp was applied for 60 minutes before visualizing the gel under ultraviolet light using a gel imaging system.

### Genotyping of E-cadherin

PCR products were Sanger sequenced using an ABI3730XL, automated DNA sequencer provided by Macrogen Corporation. Results were electronically analyzed and compared with the source sequence using the Basic Local Alignment Search Tool Program (BLAST) http://blast.ncbi.nlm.nih.gov/Blast.cgi and Geneious software https://www.geneious.com/geneious .

### Sample Size

According to a previous study, 23 the prevalence of E-cadherin SNPs is 33%. Assuming a power of 80% with a 90% confidence interval (CI), 5% α-error, and 1:1 allocation ratio, the calculated sample size was estimated at 200 ( *n* = 100 for each group). The analysis was performed using Epi Info software (version 7.2) developed by the CDC (Atlanta, Georgia, United States).

### Statistical Methods

Categorical variables were expressed as frequency and percentage, while means and standard deviation were used to describe continuous variables. Unpaired *t* -test was used to compare differences between continuous data. The associations between periodontal health or periodontitis with *E-cadherin* SNPs (genotypes and alleles) were determined using chi-square and Fisher's exact tests, respectively, together with odds ratio (OR) using wild-type genotype as a reference. The association between *E-cadherin* SNPs with stages 2 and 3 and grades B and C of periodontitis was analyzed to compare the distribution of SNPs across cases with different severities and rate of bone loss progression. Comparisons of the expected and observed genotypes were performed according to Hardy–Weinberg equilibrium (HWE). The multivariable binary logistic regression model, adjusted by age, sex, and family history, was used to determine the power of *E-cadherin* SNPs to predict periodontitis. In this model, it was assumed that exposure to SNPs (independent variables) contributed to the outcome, that is, periodontitis (dependent variable). Periodontal health and periodontitis were dichotomized by coding them as 0 and 1, respectively. Similarly, this binary coding (0, 1) was also applied to stages 2 and 3 and grades B and C, respectively. The heterozygous genotypes received code 1, while code 0 was given for the minor homozygous genotypes. The correlation coefficient ( *r* ^2^ ) between SNP loci was calculated by using the linkage disequilibrium (LD) equation. All statistical analyses were conducted by using SPSS (version 26, IBM, United States) and GraphPad Prism (version 9) considering significant differences with a *p* -value < 0.05.

## Results

After applying the eligibility criteria, a total of 829 subjects (out of 1,036) were excluded for a range of reasons. For the final analysis, 207 participants were recruited and assigned as controls (Ctrl, *n* = 105) and cases (periodontitis, *n* = 102). The mean age of the participants, self-reported family history of periodontitis, and the clinical parameters, including stage, grade, BOP, PPD, and CAL of both groups are provided in Table 1 .

**Table 1:** Demographic and clinical variables of the study population

	Ctrl	Periodontitis	*p* -Value
**Sex** a			
** Male**	63, 60.0	58, 56.9	
** Female**	42, 40.0	44, 43.1	0.7
**Age** b	38.3 ± 7.7	41.3 ± 8.3	0.05
**Clinical parameters** b			
** BOP**	0.07 ± 0.01	0.4 ± 0.2	** <0.001 c**
** PPD (mm)**	2.8 ± 0.7	4.3 ± 0.8	** <0.001 c**
** CAL (mm)**		3.5 ± 1.2	
**Stage** a			
** 2**		28, 27.0	
** 3**		74, 73.0	
**Grade** a			
** B**		54, 52.7	
** C**		48, 47.3	
**Positive family history** a			
** No**	68, 64.8	47, 45.9	
** Yes**	37, 35.2	55, 54.1	** 0.007 d**
** Total**	105, 100	102, 100	

Abbreviation: BOP, bleeding on probing, CAL, clinical attachment loss; Ctrl, healthy periodontium; PPD, probing pocket depth.

Notes: Bold font indicates significant differences.

a Frequency, %.

b Mean ± standard deviation.

c Significant difference at *p* < 0.05 by using unpaired *t* -test.

d Significant difference at *p* < 0.05 by using chi-square test.

Following DNA sequencing, *E-cadherin* SNPs, rs16260, rs10272115, rs3743674, and rs5030625, were investigated for their association with periodontal health and periodontitis ( Table 2 ). The observed frequency of the genotypes of all *E-cadherin* SNPs showed a significant difference from the anticipated distribution according to HWE except for the genotypes of the rs10272115 and rs3743674 in the periodontitis group ( Table 2 ). Heterozygous genotypes were most frequently identified in all SNPs in the study population. The genotypes and allele frequencies of rs16260 and rs10272115 *E-cadherin* SNPs were not associated with periodontal health or periodontitis. Genotypes T/T of rs3743674 and G/G of rs5030625 *E-cadherin* SNPs showed significant association with periodontitis (OR 2.783 and 3.479, respectively). In addition, the T and G alleles of the rs3743674 and rs5030625 SNPs, respectively, were significantly associated with the disease phenotype (OR 1.838 and 2.059, respectively) ( Table 2 ). Further subanalysis was performed to compare the distribution of *E-cadherin* SNPs according to stages 2 and 3 and grades B and C of periodontitis ( Table 3 ). Notably, for disease severity, the stage did not show a significant association with any *E-cadherin* SNPs ( Table 3 ). However, periodontitis grade C was significantly associated with T/T and G/G genotypes and T and G alleles of rs3743674 and rs5030625 SNP, respectively ( Table 3 ). In detail, the prevalence of the T/T genotype (62.9%) and T allele (72.9%) of rs3743674 SNP were significantly higher in grade C than in grade B periodontitis. Similarly, the G/G genotype and G allele of rs5030625 SNP were significantly higher 60 and 71.9%, respectively, in cases with a high rate of bone loss (grade C) in comparison to grade B periodontitis ( Table 3 ).

**Table 2:** Genotype and allele frequencies of *E-cadherin* rs16260 (C/A), rs10272115 (A/G), rs3743674 (C/T), and rs5030625 (GA/GA) SNPs in patients with periodontitis ( *n* = 74) and the control group ( *n* = 76)

*E-cadherin* SNPs genotypes	Ctrl a	Periodontitis a	*p* -Value b	OR	95% CI
**rs16260 (C/A)**					
** C/C c**	32, 30.3	36, 35.1		1	
** C/A**	62, 59.2	58, 56.8	0.54	0.831	0.452–1.515
** A/A**	11, 10.5	8, 8.1	0.40	0.646	0.221–1.718
*** p*** ** -Value HWE b**	**<0.001**	**<0.001**			
** Alleles**					
** C c**	126, 60.0	130, 63.7		1	
** A**	84, 40.0	74, 36.3	0.43	0.854	0.576–1.260
**rs10272115 (A/G)**					
** A/A c**	29, 27.6	19, 18.9		1	
** A/G**	59, 56.6	72, 70.3	0.07	1.863	0.934–3.697
** G/G**	17, 15.8	11, 10.8	0.99	0.987	0.362–2.513
*** p*** ** -Value HWE b**	0.62	0.55			
** Alleles**					
** A c**	117, 55.9	110, 54.1		1	
** G**	93, 44.1	94, 45.9	0.76	1.075	0.727–1.590
**rs3743674 (C/T)**					
** C/C c**	32, 30.3	24, 23.5		1	
** C/T**	51, 48.7	33, 32.4	0.86	0.927	0.471–1.857
** T/T**	22, 21.1	45, 44.1	**0.01**	2.783	1.300–5.858
*** p*** -V ** alue HWE b**	**<0.001**	0.08			
** Alleles**					
** C c**	115, 54.6	81, 39.7		1	
** T**	95, 45.4	123, 60.3	**0.002**	1.838	1.241–2.693
**rs5030625 (GA/GA)**					
** GA/GA c**	44, 42.1	19, 25.7		1	
** G/GA**	43, 40.8	28, 37.8	0.25	1.535	0.801–2.861
** G/G**	18, 17.1	27, 36.5	**0.001**	3.479	1.634–7.459
*** p*** -V ** alue HWE b**	**<0.001**	**<0.001**			
** Alleles**					
** GA b**	131, 62.5	91, 44.6		1	
** G**	79, 37.5	113, 55.4	**<0.001**	2.059	1.386–3.032

Abbreviations: CI, confidence interval; Ctrl, healthy periodontium; HWE, Hardy–Weinberg equilibrium; OR, odds ratio; SNP, single nucleotide polymorphism.

Note: Bold indicates significant differences.

a Frequency, %.

b Significant difference at *p* < 0.05 by using the chi-square test.

c Reference: wild genotype/wild type.

**Table 3:** Genotype and allele frequencies of E-cadherin rs16260 (C/A), rs10272115 (A/G), rs3743674 (C/T), and rs5030625 (GA/GA) SNPs according to stage and grade of periodontitis ( *n* = 74)

		*E-cadherin* SNPs																			
		rs16260 (C/A)					rs10272115 (A/G)					rs3743674 (C/T)					rs5030625 (GA/GA)				
		Genotypes			Alleles		Genotypes			Alleles		Genotypes			Alleles		Genotypes			Alleles	
		C/C a	C/A	A/A	C a	A	A/A a	A/G	G/G	A a	G	C/C a	C/T	T/T	C a	T	GA/GA a	G/GA	G/G	GAa	G
Stage b	S2	8, 30.0	17, 60.0	3, 10.0	33, 60.0	23, 40.0	7, 25.0	18, 65.0	3, 10.0	32, 57.1	24, 42.9	6, 20.0	8, 30.0	14, 50.0	20, 35.7	36, 64.3	7, 25.0	13, 45.0	8, 30.0	27, 48.2	29, 51.8
	S3	27, 36.5	41, 55.4	6, 8.1	95, 64.2	53, 35.8	12, 16.7	54, 73.0	8, 10.8	78, 52.7	70, 47.3	18, 24.1	26, 35.2	30, 40.7	62, 41.9	86, 58.1	19, 25.9	26, 35.2	29, 38.9	64, 43.2	84, 56.8
*p* -Value c			0.63	0.67		0.51		0.38	0.70		0.63		0.99	0.59		0.52		0.78	0.76		0.53
OR		1	0.713	0.592	1	0.801	1	1.750	1.556	1	1.197	1	1.083	0.714	1	0.770	1	0.736	1.336	1	1.222
95% CI			0.2669–1.795	0.124–2.577		0.434–1.524		0.645–4.808	0.320–6.739		0.654–2.227		0.314–3.424	0.220–2.068		0.417–1.421		0.263–2.082	0.429–3.927		0.668–2.220
Grade b	B	19, 35.9	32, 59.0	3, 5.1	70, 64.8	38, 35.1	11, 20.4	37, 68.5	6, 11.1	59, 54.6	49, 45.4	15, 28.2	25, 46.2	14, 25.6	55, 50.9	53, 49.1	18, 33.3	28, 51.3	8, 15.4	64, 59.3	44, 40.7
	C	16, 33.3	27, 56.3	5, 10.4	59, 61.5	37, 38.5	8, 17.1	35, 72.9	5, 10.4	51, 53.1	45, 46.9	8, 17.1	10, 20.0	30, 62.9	26, 27.1	70, 72.9	8, 17.1	11, 22.9	29, 60.0	27, 28.1	69, 71.9
*p* -Value c			0.98	0.45		0.66		0.30	0.98		0.88		0.77	**0.01**		**<0.001**		0.98	**<0.001**		**<0.001**
OR		1	1.002	1.979	1	1.155	1	1.782	1.146	1	1.062	1	0.750	4.018	1	2.794	1	0.884	8.156	1	3.717
95% CI			0.438–2.330	0.399–8.263		0.665–2.007		0.674–5.322	0.2388–5.047		0.609–1.853		0.231–2.152	1.319–10.70		1.526–4.903		0.304 to 2.551	2.409 to 25.56		2.034 to 6.528

Abbreviations: CI, confidence interval; HWE, Hardy–Weinberg equilibrium; OR, odds ratio; SNP, single nucleotide polymorphism.

Note: Bold font indicates significant differences.

a Reference: wild genotype/wild type.

b Frequency, %.

c Significant difference at *p* < 0.05 by using Fisher's exact test.

Based on the above-mentioned results, multivariable binary regression analysis was performed to determine the predictors of periodontitis including all SNPs adjusted according to the sex, family history of periodontitis, and age. The model was first checked if it fit the data using Hosmer–Lemeshow goodness-of-fit test which showed a *p* -value >0.05 which confirmed the significance of the model, and it was further interpreted ( Table 4 ). According to this model, the data indicated that rs5030625 and rs3743674 *E-cadherin* SNPs together with age could be used as predictors for susceptibility to periodontitis with 80.6% overall accuracy ( Table 4 ). Both *E-cadherin* SNPs showed a positive coefficient of estimate with adjusted OR 3.829 (95% CI 1.294–11.336), and 4.651 (95% CI 1.582–13.672) for rs5030625 and rs3743674, respectively, whereas the age was negatively associated with the outcome and the adjusted OR was 0.855 (95% CI 0.809–0.904) ( Table 4 ). These results suggested that the exposure to rs5030625 and rs3743674 *E-cadherin* SNPs increased the odds of periodontitis, while increasing age was associated with decreasing the likelihood of outcome in the absence of genetic variants. The same model was used for further subanalyses to determine which SNP could be used to predict stage and grade of periodontitis. For stage predictors model, all SNPs and demographic variables did not show the potential to predict the severity of periodontitis. However, grade predictors model demonstrated that rs3743674 *E-cadherin* SNP and age could anticipate the rate of bone loss with overall accuracy of 75.7% ( Table 4 ). The coefficient of estimate for rs3743674 *E-cadherin* SNP and age were 1.426 and −0.053, respectively. This indicated that increasing the age can be considered as a protective factor (OR 0.948, 95% CI 0.902–0.997) against progressive rate of alveolar bone loss, while the risk of rapid bone loss in periodontitis patients was potentially increased up to four times in the presence of rs3743674 *E-cadherin* SNP ( Table 4 ).

**Table 4:** Accuracy of *CDH1* SNPs to predict periodontitis, stage, and grade by using a backward binary logistic regression model

SNP	B	SE	*p* -Value a	Exp (B)	95% CI		Accuracy of the model		
					Lower	Upper	Sensitivity	Specificity	Overall accuracy
**Periodontitis predictors**									
** rs16260**	−0.049	0.476	0.92	0.952	0.374	2.422	85.1%	76.3%	80.6%
** rs10272115**	0.337	0.477	0.48	1.400	0.549	3.568			
** rs3743674**	1.537	0.550	**0.005**	4.651	1.582	13.672			
** rs5030625**	1.343	0.554	**0.01**	3.829	1.294	11.336			
**Sex (male)**	−0.446	0.455	0.33	0.640	0.262	1.563			
**+ family history**	0.554	0.478	0.25	1.740	0.682	4.440			
**Age**	−0.157	0.028	**<0.001**	0.855	0.809	0.904			
				Hosmer–Lemeshow test b			Chi-square, df 4.6, 8	Sig. 0.79	
**Stage predictors**									
** rs16260**	1.099	0.577	0.06	3.000	0.968	9.302	91.8%	24.0%	68.9%
** rs10272115**	−0.808	0.621	0.19	0.446	0.132	1.505			
** rs3743674**	0.545	0.675	0.41	1.725	0.459	6.481			
** rs5030625**	0.048	0.604	0.93	1.050	0.321	3.429			
**Sex (male)**	0.268	0.523	0.60	1.308	0.469	3.645			
**+ family history**	−0.028	0.604	0.96	0.972	0.297	3.179			
**Age**	0.003	0.024	0.91	1.003	0.957	1.051			
				Hosmer–Lemeshow test b			Chi-square, df 8.3, 8	Sig. 0.34	
**Grade predictors**									
** rs16260**	0.382	0.625	0.54	1.466	0.431	4.989	70.3%	81.1%	75.7%
** rs10272115**	−0.288	0.691	0.67	0.749	0.193	2.903			
** rs3743674**	1.426	0.701	**0.02**	4.161	1.071	9.797			
** rs5030625**	0.757	0.613	0.21	2.131	0.641	7.089			
**Sex (male)**	0.961	0.558	0.08	2.615	0.876	7.805			
**+ family history**	−1.419	0.826	0.09	0.241	0.047	1.221			
**Age**	−0.053	0.025	**0.03**	0.948	0.902	0.997			
				Hosmer–Lemeshow test b			Chi-square, df 7.1, 8	Sig. 0.52	

Abbreviations: B, coefficient of estimate; CI, confidence interval; Exp (B), adjusted odds; SE, standard error; SNP, single nucleotide polymorphism.

a Significant difference at *p* < 0.05 is indicated by a bold font.

b Goodness-of-fit test of the model.

Frequencies of haplotypes are illustrated in Table 5 . In total, 11 haplotypes were determined but 2 haplotypes were not present in healthy controls (GGAT and GAGCC) and the same applied to the periodontitis group (GAAC and GGAC). To determine the nonrandom association between different variants, LD analysis was conducted. The results indicated the presence of a close correlation between rs5030625 and rs3743674 *E-cadherin* SNPs ( *r* ^2^ = 0.638) in the disease phenotype indicating a disequilibrium state between these variants. Similarly, a moderate correlation ( *r* ^2^ = 0.495) was observed between rs10272115 and rs16260 variants ( Table 6 ).

**Table 5:** Frequency distribution of *E-cadherin* haplotypes between healthy control and periodontitis groups

Haplotypes	Ctrl a	Periodontitis a	Total
**GAAC**	4, 2.0	0, 0.0	4, 1.9
**GAAT**	3, 1.3	3, 1.3	6, 2.9
**GACT**	26, 12.7	34, 16.6	60, 29.0
**GGAC**	4, 2.0	0, 0.0	4, 1.9
**GGCC**	6, 2.7	3, 1.3	9, 4.3
**GGCT**	6, 2.7	6, 2.7	12, 5.8
**GGAT**	0, 0.0	1, 0.7	1, 0.5
**GAACC**	54, 26.0	29, 14.0	83, 40.1
**GAACT**	1, 0.7	15, 7.3	16, 7.7
**GAAAC**	1, 0.7	10, 4.7	11, 5.3
**GAGCC**	0, 0.0	1, 0.7	1, 0.5
**Total**	105, 50.7	102, 49.3	207, 100

Abbreviation: Ctrl, healthy periodontium.

a Frequency, %.

**Table 6:** Correlations between *E-cadherin* variants loci

Loci	rs16260	rs10272115	rs3743674	rs5030625
**rs16260**		**0.495**	0.023	0.087
**rs10272115**	**0.495**		0.001	0.011
**rs3743674**	0.023	0.001		**0.638**
**rs5030625**	0.087	0.011	**0.638**	

Notes: Correlations expressed as correlation coefficient ( *r* ^2^ ). Significant correlations are indicated by a bold font.

## Discussion

Sequencing analysis of DNA from blood samples from patients with periodontitis indicated that alleles and genotypes of rs5030625 and rs3743674 *E-cadherin* SNPs were significantly associated with periodontitis. The lack of equilibrium was also observed between these loci. Additionally, these SNPs exhibited the potential to provide good prediction of periodontitis disease levels. These results supported the rejection of the null hypothesis and were consistent with cumulative evidence supporting the important role of genetic factors which result in increasing susceptibility to periodontitis. 10 To the best of our knowledge this is the only study to have investigated the association between *E-cadherin* SNPs in periodontitis pathogenesis.

Millions of SNPs in the human genome have been identified, approximately half of them affect noncoding regions, while others lead to missense and silent (synonymous) mutations and are therefore responsible for pathologies by modulating promoter activity and pre-mRNA stability. 24 Consequently, the role of these gene variants in the predisposition of disease is in response to certain environmental cues. Indeed, exposure to periodontal pathogens of a dysbiotic microbiome is the main driver for triggering periodontal disease. 6 In addition, an individual's susceptibility or resistance to disease progression and severity are highly determined by other behavioral, environmental, and genetic factors. Notably, all steps of gene expression can be significantly altered when genetic variation occurs and certain genomic locations can be affected. 25 For instance, noncoding regulatory SNPs can modify the level of gene transcription by altering the methylation of the promoter sites, mRNA splicing, protein activity, and translation. 25 Interestingly, epigenetic events such as DNA hypermethylation of CDH1-CpG islands and subsequent silencing of *E-cadherin* are associated with periodontitis, resulting in irreversible tissue destructions in a similar manner to that of breast cancer. 26 Results of a systematic review previously concluded that the crosstalk between SNPs and epigenetic risk factors associated with allele-specific gene expression is a major contributor to the pathogenic mechanism of many multifactorial diseases. 27

Previous work using gingival biopsies collected from patients with periodontitis has indicated that decreased E-cadherin expression, in comparison with healthy tissue, is a key pathological event in epithelial pocket lining which could be responsible for loss of tissue integrity and spread of bacteria to deeper tissues. 15 Interestingly, susceptibility to epithelial-mesenchymal transition (EMT), which is a process causing loss of epithelial phenotype in many diseases, including periodontitis is mediated by certain gene variants that modulate *E-cadherin* transcriptional activity. 28 Previously, a novel C/A polymorphic site was identified, specifically at the −160 location, of the *E-cadherin* promoter in cancer specimens which exhibited aberrant transcriptional activity. 29 Subsequently, a relatively large number of studies, including the involvement of many ethnic groups, has highlighted a strong association of the regulatory *E-cadherin* −160C/A SNP with malignant and nonmalignant diseases. 18 The current study did not show any association between genotypes and alleles of rs16260 and periodontitis; however, this could be attributed to the sample size and ethnic factor. One of the genotypes of *E-cadherin* rs3743674 SNP studied here was significantly associated with the periodontitis phenotype and the T allele of this SNP was associated significantly with rapidly progressive (grade C) periodontitis. No relevant literature linking this *E-cadherin* SNP with periodontitis has been identified; however, some studies showed a significant association between *E-cadherin* rs3743674 SNP with breast 30 and gastric cancer 31 in populations with different ethnic backgrounds. Similarly, the G/G genotype and G allele of rs5030625 did demonstrate associations with phenotype and grade of periodontitis. Notably, a previous study suggested that −347G→GA could be responsible for compromised E-cadherin expression by affecting the binding affinity of the transcriptional factor and reducing transcriptional activity. 32 Changes in levels and functionality resultant from *E-cadherin* variants could be responsible for a more rapid progression of periodontitis. Changes in the E-cadherin molecule could potentially compromise keratinocyte coherence and increase susceptibility to epithelial breakdown via impaired barrier function due to relatively minor local tissue disruptions. Similar disease mechanistic processes are proposed in patients with Crohn's disease in which *E-cadherin* SNPs have identified increased epithelial permeability due to abnormal cytoplasmic accumulation and impaired cell membrane localization of E-cadherin. 33 Consequently, any further infection and inflammation would lead to the ingress of periodontal pathobionts into the underlying tissues, which would lead to exacerbating effects on E-cadherin levels/function and loss of epithelial phenotype via EMT. This would likely lead to a chronic disease cycle with local tissue healing being impaired.

Multivariate regression model indicated that age, besides *E-cadherin* SNP, could accurately predict susceptibility to periodontitis. This predictor was negatively associated with the outcomes which suggest that increasing age is a protective factor against any deleterious effect of genetic variants. The susceptibility genotype contributes more for inducing early onset and severe phenotypes of periodontitis, formerly known as aggressive periodontitis, in younger subjects, indicating that disease onset is genetically predisposed. 34 The influence of genetic factors appears to decrease in the moderate phenotype of periodontitis in older subjects and this could be due to increasing senescence of the immune system during aging and/or the effect of lifestyle and environmental factors. 34 These data were consistent with results from this study in which *E-cadherin* SNPs were significantly associated with the severe and rapidly progressing form of periodontitis (grade C) which is more evident in younger age groups. 1 Other results further supported this notion which indicated that *E-cadherin* rs3743674 SNP and age could predict the progression rate of periodontitis. The purpose of introducing grade of the disease into the latest classification system of periodontal diseases/conditions was to complement clinical staging of periodontitis which reflect its severity/extent with information derived from inherent biological grade of periodontitis. 1 This could explain why *E-cadherin* SNPs did not show any potential to predict stage of periodontitis which is a pure clinical indicator of severity relying mainly on measuring interdental CAL and radiographic bone loss. 1 These clinical parameters represent instantaneous measurements of the cumulative destruction of periodontal tissues resulted from previous exacerbations of periodontitis which is a disease of episodic nature regulated by several local and systemic factors together with genetic. 35

Results from this study showed that a positive family history of disease was significantly associated with periodontitis; however, these far data have not been provided to accurately predict periodontitis pathogenesis. Furthermore, periodontitis is a complex disease of multietiology that does not follow a simple pattern of genetic transmission. 36 Identification of biomarkers for predicting, diagnosing, and monitoring periodontal diseases is now receiving increasing attention. 37 Molecules available in oral fluids or tissues are either derived from the host, inflammatory and genetic biomarkers, or of bacterial origin. 38 The new classification system of periodontal/peri-implant diseases is now providing a revolutionary attempt to integrate available clinical and biological evidence in diagnosis. Defining the severity and anticipating the rate of progression are integral components of the new classification system. 1 However, calculations of these domains depend entirely on conventional periodontal charting using manual probing, supported with radiographic information, and this remains the gold standard for diagnosing periodontal diseases. These techniques could be subjected to iatrogenic or inherited discrepancies and cannot define the history of destruction or predict the onset of the disease which may compromise the diagnosis and treatment plan. 39 Although the inclusion of biomarkers in the new classification scheme was recommended, this concept is still far from realistic in application in routine dental practice until available biomarkers become thoroughly validated. The best example of using genetic biomarkers to identify subjects at risk of developing periodontitis is the IL-1-based commercially available genetic susceptibility test. The basis of this assay relies on the simultaneous occurrence of an SNP at the IL-1A + 4845 and IL-1B + 3954 loci which result in excessive IL-β production in response to lipopolysaccharide from gram-negative periodontal pathogens. 40 In a similar context, LD analysis in this study showed that rs3743674 and rs5030625 *E-cadherin* SNPs, significantly associated with periodontitis, were nonrandomly correlated with each other. Additionally, regression analyses indicated that the accuracy of the above-mentioned *E-cadherin* SNP as predictors of periodontitis was about 80%.

This study identifies the first attempt to investigate the association between *E-cadherin* SNPs with periodontitis in the Iraqi population. Notably, certain limitations could restrict the generalization of our results. Conducting further studies with a larger sample size and extending stratification of the sample according to independent demographic and clinical variables together with including all stages and grades of periodontitis are recommended. In addition, further serological and microbiological analyses, and inclusion of other ethnic groups are required to further clarify the role of *E-cadherin* SNPs in the pathogenesis of periodontitis. Furthermore, a causal relationship between *E-cadherin* SNPs and periodontitis is yet to be determined. Consequently, however, the findings of this study represent a novel opportunity to identify genetic variants that could be responsible for modulating the host response, this may aid our better understanding of the pathogenic mechanism involved, facilitate early diagnosis, and ultimately enable development of novel targeted treatment modalities.

## Conclusion

The genotypic and allelic variants of *E-cadherin* , particularly rs3743674 and rs5030625, were associated with periodontitis, particularly with grade C. Consequently, these biomarkers may exhibit predictive potential for this disease. Additionally, *E-cadherin* rs3743674 SNP possessed the ability to predict the grade of periodontitis. This suggested that the genetic makeup of the host may influence *E-cadherin* expression when exposed to the disease's drivers and risk factors. Further studies are recommended to elucidate the role of this protein and its variants in the pathogenesis of periodontitis and enable justification of its role as a predictor for disease susceptibility.
